# In Defense of Applied Behavior Analysis and Evidence-Based Practice

**DOI:** 10.1007/s40614-025-00468-y

**Published:** 2025-08-18

**Authors:** Jason Travers, Matt Tincani

**Affiliations:** 1 Department of Teaching and Learning, Temple University, Philadelphia, PA USA; 2 Department of Psychological Studies in Education, Temple University, 1301 Cecil B. Moore Avenue, Philadelphia, PA 19122 USA

**Keywords:** Ableism, Applied behavior analysis, Autism spectrum disorder, Evidence-based practice, Developmental disabilities, Pseudoscience, Neurodiversity, Trauma-informed practice

## Abstract

This commentary critically appraises attacks on applied behavior analysis (ABA) from outside and—increasingly—within the field. Commonly repeated attacks are that ABA is coercive and suppresses individual identity, aligns with the medical model, causes trauma, and, in more extreme cases, constitutes abuse. We illustrate how these claims are based on unfounded criticism and longstanding myths about ABA and stand in direct contrast to the empirical foundations of behavior analysis. We also highlight how such criticism conflicts with over half a century of evidence that ABA supports autonomy and enhances wellbeing of people with autism and developmental disabilities. We call for self-reflection among well-meaning behavior analysts who repeat such criticisms and greater attention to evidence-based practice.

Over 50 years of applied behavior analysis (ABA) research has led to the development of knowledge about how to change behavior in ways the person (or their caregivers) value (Heward et al., 2022; Tincani et al., 2024). Although based on scientific evidence, values held by recipients, professionals, and communities inform intervention goals, what interventions are used, and how interventions are applied. Indeed, a core tenet of ABA is prioritizing socially significant changes in a person’s behavior (Baer et al., 1968). ABA research and practice has been met with resistance, however, and much it can be traced to deeply held beliefs about the sources of behavior and rules that govern it (e.g., divinely revealed morality, punitive-based justice/legal systems, personal responsibility and internal locus of behavioral control; see Axelrod, 1996). These and other similar beliefs have negatively influenced perceptions about ABA by social scientists and the public, fueling continued dissemination of myths and misconceptions (e.g., Anderson, 2023; Slater, 2005; Stahmer et al., 2024).

ABA is not the only example of scientific knowledge and technology that has been met with criticism based on deeply held beliefs. Darwin’s theory of speciation, evolution by natural selection, has been attacked on the basis that it conflicts with religious beliefs typically associated with creationism (Miller et al., 2006, 2022). It perhaps should come as little surprise that Skinner’s theory of behavior, selection by consequences, also is met with similar hostility. Myths and misperceptions about ABA permeate traditional popular sources as well as social media and related phenomena (e.g., influencers, content creators). The perpetuation of old and new myths and misperceptions of ABA has reached seemingly vast audiences who may discourage consumers from seeking effective behavior-analytic services in schools and community-based programs (Malkin et al, 2024; Mullins et al., 2025). For some families and professionals, misleading information about ABA may instead motivate them to consider low-value and potentially harmful “alternative” approaches with insufficient evidence (e.g., dietary supplements; sensory integration treatment, spelling to communicate/facilitated communication; Travers et al., 2016).

Perusal of various social media reveals an environment teeming with familiar claims and accusations about ABA (e.g., that it is inherently coercive, prioritizes normalization, is dehumanizing), and that ABA professionals share common practices that are innately harmful to children (e.g., Direct Instruction, practice-based fluency, discrete trial teaching; Sandoval-Norton & Shkedy, 2019; see Gorycki et al., 2020, for a response to such criticism). Of course, many of the claims are sweeping generalizations that lack credible evidence and likely reflect the echoing effects of proprietary algorithms that propagate social media posts to generate user engagement. We have observed two concerning trends about these and related claims about ABA. First, social media posts from organizations and influencers repeatedly claim ABA therapy is criminally abusive and its professionals are engaged in genocide of autistic people. Although some may see such claims merely as hyperbolic clickbait unworthy of attention, such accusations without evidence have serious implications. Second, the influence of such unfounded claims has begun emanating from within the ABA community and in ways that appear inconsistent with the fundamental science and practice of ABA.

The goal of this commentary is to highlight these two concerns as threats to evidence-based practice for individuals with autism spectrum disorder (ASD) and other developmental disabilities (DD). We highlight fallacious tactics used to promote myths and disinformation, then briefly explore the potential implications for such attacks. Our intention is to highlight how evidenced-based interventions grounded in the science of ABA are fundamentally humanistic and thus incompatible with the accusations. We propose that repeating such criticism misdirects families away from effective interventions while simultaneously hindering attempts to root out ineffective interventions and malpractice. We include discussion of the ABA community, from which some of the accusations have recently begun to emanate, to illustrate the differences between advances in professional practice and those that are ill-informed. There we explore how values for evidence-based practice should also guide thought and self-evaluation of performance to address problems of social significance.

## Fallacious and Unsupported Criticism of ABA

### The Neurodiversity Movement

Online communities aligned with the neurodiversity (ND) movement often promote ill-informed and fallacious criticism of ABA. ND is based on ideas of human variation—that human neurological development naturally varies, and that people with ASD, attention deficit hyperactivity disorder, learning disabilities, or other conditions experience the world differently but not incorrectly (Leadbitter et al., 2021). From this perspective, approaches like ABA that attempt to ameliorate these conditions are not regarded as forms of help or support, but as fundamental challenges to individual identity (e.g., Shyman, 2016). Instead, many ND proponents argue that effort should focus on validation of identity, advocacy, and greater acceptance of disabled individuals by societal institutions, including accommodations and inclusion (Jaarsma & Welin, 2012).

There is considerable benefit to a perspective that values individual experience, accommodations to support greater inclusion, and consideration of strengths as well as skills in need of development. Indeed, many commonly employed ABA strategies—preference assessments, choice making, augmentative and alternative communication, and strength-based interventions, to name a few—reflect values consistent with those promoted by members of the ND movement (Prince et al., 2023; Tincani et al., 2024). However, ND critics of ABA have ignored how ABA produces outcomes consistent with shared values and instead accuse ABA professionals of coercing autistic individuals into behaving normally or otherwise causing them irreparable harm (e.g., Anderson, 2023). The lack of credible evidence to support such claims (Leaf et al., 2022; Vyse, 2022) means criticism often depends on longstanding myths about ABA. Such myths are not only used to attack professionals but also affect parents, children, and families who have benefited from ABA. Amy Lutz (2025), a bioethicist and parent of an adult with severe autism, highlighted that members of the ND community who criticize ABA often presume to speak for all individuals on the spectrum, including those with severe forms of autism. She emphasized how individuals with severe ASD, who historically have benefited from ABA, are marginalized by anti-ABA activists within the ND community who seek to curtail access to evidence-based ABA interventions.

Despite the lacking evidence and dishonest tactics to attack ABA, some ABA experts have argued that professionals must integrate a ND perspective into their practice. For example, Mathur et al. (2024) explained that ND proponents express concern that ABA therapy is harmful because it prioritizes socially acceptable behavior in ways that undermine identity development and formation of the individual. That is, Mathur et al. explained that ND proponents view efforts to reduce behavior such as excessive stereotypy in children with ASD and DD as inherently ableist. Mathur et al. further explained that ND proponents believe abusive practices are often only recognizable to autistic people, and that ABA can lead to social isolation, mental health conditions, and suicidality. Mathur et al. do not cite scientific evidence for these claims but instead point to anecdotes from social media sites and influencers who have attacked parent advocates of individuals with severe ASD as well as ABA researchers who criticize dangerous practices like facilitated communication (also called spelling to communicate and rapid prompting method). Mathur et al. also quoted the Autistic Self-Advocacy Network’s (ASAN) claims that ABA denies dignity of and compassion for autistic people, and that functional approaches to behavior are dehumanizing. It should be noted that ASAN has a long history of anti-ABA activism and support for unproven and dangerous techniques like facilitated communication (ASAN, 2011, 2018). Mathur et al.’s sources also included other social media sites where ABA is described as abusive and based on a genocidal eugenics agenda that intends to eliminate autism and autistic people. Amplifying and repeating such false claims based on anecdotes undermines ABA and likely curtails access for those most likely to benefit from these services.

### ABA and “The Medical Model”

Critics often repeat that ABA is grounded in a medical model and therefore harmful (Kirkham, 2017). This criticism is so pervasive that it is now espoused by some behavior analysts (e.g., Allen et al., 2024). Kapp et al. (2013) asserted, “The medical model aspires toward normalization, symptom reduction, and elimination of conditions identified based on deficits said to cause functional impairment in major life activities” (p. 59). It is argued the medical model of disability is problematic because it pathologizes a person’s disability, sourcing the cause of a person’s impairments within the individual (e.g., abnormal neurological functioning that is an innate characteristic of the person) rather than in the social and institutional barriers that manifest discrimination.

Changes in state laws requiring medical insurance to pay for ABA services dramatically increased access to services for children and families in the United States (Lyon et al., 2020). These reforms may also have resulted in some ABA providers adopting undesirable aspects of the medical model, such as “prescribing” ABA to eliminate challenging behavior without also focusing on skills-based instruction. However, characterizations of ABA as the medical model are problematic in several ways. First, despite frequent treatment of the medical model as an actual model of clinical practice in the disability studies literature (e.g., Shyman, 2016), there are currently few, if any, actual communities of practice that self-describe as following the medical model. To wit, criticisms of the medical model are now decades old (Brisenden, 1986), and medical professionals have shifted from their traditional model (i.e., pathologizing, curing) towards prevention-based treatment (e.g., primary, secondary, and tertiary prevention; Willis et al., 2022). Likewise, we find no evidence that ABA is grounded in a medical model or that ABA professionals rely on it to inform their practice.

Behavior analysts have long argued that behavior is the product of environment rather than some innate defect in need of correction. In particular, behavior analysts view behavior as a dynamic confluence of phylogeny (i.e., the evolutionary history of the species) and ontogeny (e.g., the reinforcement history of the individual), where selection by consequences is the causal mode (Skinner, 1966). The medical model posits that disabilities are exclusively biological conditions to be diagnosed and cured, discounting the role of environmental (i.e., social) influences. This view is anathema to the decades-long behavior-analytic conception of behavior, which emphasizes careful analysis of the environment, and social validation of intervention goals, procedures, and effects (Schwartz & Baer, 1991; Skinner, 1953).

Moreover, irrespective of professionals’ ontological perspective about the origins of disabilities and associated impairments, in some cases medical intervention may benefit individuals with ASD and DD. For example, some pharmacological treatments change physiological or biological function in ways that may reduce the frequency and intensity of severe behavior (Iffland et al., 2023). Consider that phenylketonuria, a metabolic disorder that causes intellectual disability if the person consumes food containing a specific enzyme, was virtually eliminated from the developed world through universal infant medical screening (Brosco & Paul, 2013). Seldom are these benefits acknowledged when criticizing a medical approach to intervention for individuals with DD.

### ABA is Abuse and Causes Trauma

Behavior analysts played a major role in bringing about deinstitutionalization and community inclusion. During the late 19^th^ and early to mid-20^th^ century, individuals with ASD and DD were routinely warehoused in state institutions with limited or no basic care, support, or services. They often had needs that went untreated and were otherwise mistreated, neglected, and abused, living without adequate clothing, proper nourishment, and basic comfort (e.g., beds, warmth). The abhorrent conditions of institutions were partly due to societal perceptions that people with DDs were unfeeling, subhuman organisms (Wolfensberger, 1972). Behavioral intervention research during the 1960s and 1970s showed people with developmental and other disabilities could learn valuable skills, reduce socially unaccepted or otherwise harmful behavior (e.g., physical aggression, self-injury), participate in the community, and experience overall greater well-being (Kazdin, 1978; see also Morris et al., 2013, for a summary history of early ABA research for individuals with ASD and DD). Despite behavioral research that contributed to the end of systematic abuse and trauma of people with disabilities in state institutions, ABA services have been characterized as abusive and traumatizing in ways that may discourage families from seeking evidence-based behavioral interventions and supports.

Critics often point to research by Lovaas conducted in the 1970s and 1980s, erroneously referring to him as “the father of ABA” (Elias, 2025) or “the founder of ABA” (Gibson & Douglas, 2018) despite being only one of many historical figures associated with the early history of the field (Morris et al., 2013). Lovaas used corporal punishment to reduce behavior, though he later disavowed these techniques (Smith & Eikeseth, 2011). Despite significant advances in applied intervention research and practice, the historical use of punishment to modify behavior is often used to condemn current ABA as abusive and traumatizing. The proposition, it seems, is that contemporary ABA is inextricably entwined with past practice (Johnson, 2022). The implication is that all ABA professionals use corporal or other punishment procedures and thus cause clinical trauma to individuals they serve. This is, in our view, a fallacious criticism. ABA professionals are bound by professional ethics to abstain from corporal punishment and instead must rely on reinforcement-based interventions and only use more restrictive interventions when it is in the consumer’s best interest (Behavior Analyst Certification Board [BACB], 2020).

It is important to remember that the claim that “ABA is abuse” implies that professionals are criminal abusers. This attack is also buttressed by 50-year-old studies that used punishment to change behavior and, accordingly, is a dishonest representation of contemporary practice. Consider by analogy that early medical practitioners employed an array of dangerous and deadly practices that frequently harmed or killed patients including bloodletting, using unsterilized medical instruments, and crude amputation without anesthesia (Rutkow, 2023). We do not, however, condemn modern day oncologists who administer chemotherapy as barbaric professionals who impose traumatizing experiences on the individuals they serve based on practices in the distant past. Instead, we acknowledge that modern medicine has progressed, and that historical practices illustrate the dangers of human ignorance, the value of scientific knowledge, and the progression of science. Current ABA professionals should not be condemned or alleged to be abusers based on the harmful practices that were abandoned decades ago. Nevertheless, the notion that ABA is abusive, and its practice trauma-inducing, appears as a popular trope in social and popular media.

Trauma-informed practice has found a home in ABA (e.g., Austin et al., 2024; Rajaraman et al, 2022), despite a lack of compelling evidence (e.g., Maynard et al., 2019). Critics have falsely asserted ABA causes trauma (e.g., Kupferstein, 2018; see Leaf et al., 2018 for a response). Acceptance and adoption of trauma-informed practice within ABA risks reifying this claim without careful consideration of the nature of trauma and the evidence in support of trauma-informed practice. The *DSM-V* defines posttraumatic stress disorder (PTSD) as “exposure to actual or threatened death, serious injury, or sexual violence” (American Psychiatric Association, 2013). PTSD can occur through directly experiencing traumatic events, directly witnessing others experience traumatic events, learning that traumatic events occurred to family members or close friends, or through repeated exposure to the details of traumatic events, such as by first responders to natural disasters.

Kranak and Briggs (2025) highlighted how advocates of trauma-informed practice in ABA discount real trauma experienced by people with PTSD by mischaracterizing all aversive events as “traumatic.” They described how everyday events encountered in ABA assessment and intervention (e.g., removal of preferred stimuli, presentation of instructional requests) are not trauma-inducing according to well-established definitions but are necessary components of assessment and intervention. Some ABA professionals appear to believe that benign procedures used in nearly all educational and treatment contexts can cause trauma. Such perceptions undermine the impact of evidence-based interventions by misleading professionals away from established assessment methods, interventions and related procedures, and potential learning opportunities.

Curiously, although advocates have enthusiastically touted the benefits of trauma-informed practices in ABA, they have also acknowledged lack of a coherent behavior-analytic definition of trauma and a lack of evidence in support of trauma-based approaches. For example, in their article advocating for trauma-informed care (TIC) in ABA, Austin et al. (2024) conceded, “we do not know specifically the types of stimuli or interactions that will produce traumatic stress or other negative health outcomes, for whom, and under what conditions” (p. 670). They further conceded, “TIC as a unified approach currently lacks evidence” (p. 674). Despite acknowledging these shortcomings, they contended behavior analysts should “align their services with TIC by designing services that acknowledge the impact of trauma, foster safety and trust, promote choice and shared governance, and focus on skill building” (p. 671).

It should be noted that components of clinical practice such as fostering trust, promoting choice and shared decision-making, and facilitating skill building are not exclusive to TIC—these often are understood as necessary components of any sound approach to clinical treatment (e.g., Coyne, 2008), including ABA (Kranak & Briggs, 2025). It is thus unclear what a TIC perspective contributes beyond these well-established features. Furthermore, ABA professionals who do not self-describe their practice as trauma-informed may be unjustly characterized as engaging in trauma-uninformed practice, or even trauma-inducing practice. Given there are no objective criteria for demarcating trauma-informed and trauma-uninformed practice, one wonders whether TIC is merely a commitment to ambiguity for the sake of marketing one’s services. More fundamentally, an incomplete understanding of the construct of trauma, elements of trauma-informed practice, and benefits of trauma-informed practice in ABA should give one serious pause about integrating them into one’s professional practice until far more scientific evidence is available.

## Adhering to an Evidence-Based Practice of ABA

This commentary highlights unfounded criticisms of ABA, including that ABA is coercive and suppresses individual identity, aligns with the medical model, causes trauma, and, in more extreme cases, constitutes abuse. These and similar attacks on ABA, in our opinion, reflect limited understanding about the science of behavior, its underlying philosophy, and applications of evidence-based behavior interventions and supports to improve the lives of others. We struggle to find valuable guidance in such strident criticisms of ABA, particularly those emanating from the online ND community. However, we believe that ABA professionals for whom such criticisms resonate, overall, have the best interests of their consumers in mind, as they believe these perspectives will help improve outcomes for individuals and families. Given our shared values, we offer the following considerations to maintain adherence to evidence-based practice.

## Social Validity Does Not Imply Unconditional Acceptance

Social validity refers to the value of treatment goals, procedures, and outcomes by individuals, stakeholders, and society—for over 50 years it has been a fundamental value of ABA (Baer et al., 1968; Schwartz & Baer, 1991). However, social validity does not mean unconditionally accepting the viewpoints and perspectives of any self-identified group as inherently valuable or true, especially when those viewpoints are misinformed and/or serve to disinform. We agree with Lutz’s (2025) perspective that shared opinions of the ND community do not and cannot represent the needs, interests, strengths, preferences of all individuals with ASD. Still, some behavior analysts have suggested that affirming criticisms of ABA is not only compassionate and ethical, but also necessary for acceptance of our approach (e.g., Allen et al., 2024; Mathur et al., 2024).

We contend that unconditionally embracing the ND perspective, or any ontological perspective, risks falsely validating and reifying unfounded criticisms of evidence-based ABA and further undermining the credibility of the science and practice of behavior analysis. Moreover, resulting features of “ND affirming practice” may result in avoidance of effective teaching strategies and/or presentation of fewer learning opportunities, for fear that professionals are perpetuating forms of ableism and abusing the people they serve. Such an approach likely hinders access to evidence-based ABA and fuels further cynicism and rejection of ABA by those who would otherwise benefit from it.

### Embracing Universalism

Influential sociologist of science Robert K. Merton (1973) identified universalism as one of the fundamental norms of science. Universalism “finds immediate expression in the cannon that truth-claims, whatever their source, are subjected to *preestablished impersonal criteria*; consonant with observation and with previously confirmed knowledge.” (p. 270). That is, scientists, and society at large, should resist allowing individual identities and personal values to influence appraisal of truth claims and instead rely on objective observation and confirmed knowledge (i.e., knowledge derived from rigorous, unbiased research). He described the deleterious consequences that result “when the larger culture opposes universalism” (p. 271), such as when social and political movements undermine scientific inquiry and knowledge generation. One of the most notorious historical examples is proliferation of Lysenkoism, a pseudoscientific approach to agriculture aligned with the ideological beliefs of Soviet communism, which rejected genetic science. Lysenkoism resulted in ineffective farming methods in the Soviet Union that caused a major famine and death by starvation of millions of people (Borinskaya et al., 2019).

Likewise, facilitated communication is an ineffective and dangerous technique promulgated by rejecting dozens of sound experimental studies that show facilitators author messages and not the person with DD and communication impairment. Facilitated communication is the single most discredited technique in the history of DD (Mostert, 2001, 2010; Schlosser et al., 2014). Like proponents of Lysenkoism who rejected well-understood scientific principles of genetics, prominent ND organizations vehemently defend facilitated communication while simultaneously attacking ABA with appeals to emotion, equivocation, ad hominem, and other fallacies. Facilitated communication is understood by most members of the ABA community to be ineffective and even harmful. Behavior analysts should strongly question the validity of anti-ABA sentiments (e.g., ABA is harmful, abusive) given there is no evidence for such claims, especially when sourced from individuals and groups who promulgate unproven, disproven, and even harmful techniques like facilitated communication (ASAN, 2018), bleach therapy (Association for Science in Autism Treatment, n.d.), and various homeopathic remedies purported to “cure” autism (Boseley, 2018).

## Relying on Science and Evidence-Based Practice

Behavior analysts embrace a scientific perspective of behavior. That is, they rely on direct evidence when available and conduct careful, well-planned, and controlled analyses of behavior to determine functional relations with the environment. All claims about ABA (and any clinical approach) should be subjected to the same standards of scientific inquiry and skepticism. There is no a priori justification for preferencing certain claims over others. Therefore, when confronted with claims that ABA devalues autistic identity, aligns with the medical model, or is abusive, behavior analysts should ask, “What is the evidence for this claim?” just as they should when confronted with claims that any intervention should be used to address an individuals’ needs. This perspective may seem axiomatic to many in the field but given increasing proliferation of anti-ABA sentiment within ABA and beyond, it is important to emphasize the significance of an evidence-based approach to decision making.

The ABA profession has grown at an astonishing pace in recent years, with the number of BACB certified professionals approximately doubling between 2019 and 2025 (BACB, 2025). Although this growth has resulted in increased access to ABA services for individuals and families, an undoubtedly positive development, it also has produced a large cadre of early-career professionals who have limited experiences and varying qualities of scientific training. Given proliferation of continuing education workshops, conferences, and professional development activities that perpetuate anti-ABA sentiments and/or promote ND affirming practices, we are concerned that professionals may presume the content of such offerings is evidence-based and informs ethically sound practice, especially with the imprimatur of a credentialing organization. Moreover, relatively inexperienced and nonscientifically trained professionals may be ill equipped to critically appraise the content of trainings and claims made by presenters who appear to be reliable sources due to their reputation and credentials. We therefore echo recent calls for greater attention to and reliance on scientific evidence about the effects of ABA, and that professionals adhere to evidence-based practice in professional development aligned with the values of our science (Boggs et al., 2025; Critchfield, 2024; Rapp et al., 2025).

## Data Availability

There are no data associated with this article.
